# Association of Short-Course Methylprednisolone Therapy With Early Composite Imaging Abnormalities in Patients With Early Neurological Deterioration After Endovascular Thrombectomy for Acute Ischemic Stroke

**DOI:** 10.31083/RN54438

**Published:** 2026-09-22

**Authors:** Changsi Ai, Zuolei Zhu, Langqin Zhang

**Affiliations:** ^1^Department of Neurology, The Hongda Hospital of Jiamusi University, 154000 Jiamusi, Heilongjiang, China; ^2^Department of Neurosurgery, The First People’s Hospital of Xiantao, Affiliated Hospital of Hubei University of Science and Technology, 433000 Xiantao, Hubei, China; ^3^Department of Thoracic Surgery and Department of Brain Surgery, Hangzhou Red Cross Hospital (Zhejiang Hospital of Integrated Traditional Chinese and Western Medicine), 310003 Hangzhou, Zhejiang, China

**Keywords:** ischemic stroke, thrombectomy, methylprednisolone, brain edema, clinical deterioration

## Abstract

**Background:**

Early neurological deterioration (END) after endovascular thrombectomy (EVT) may be accompanied by heterogeneous postprocedural imaging abnormalities. We investigated the association between short-course methylprednisolone and a study-defined early composite imaging abnormality after EVT.

**Methods:**

This single-center retrospective analysis included 297 patients with acute ischemic stroke who developed END within 72 hours after EVT (96 treated with methylprednisolone and 201 not treated). The index time was defined as completion of the post-END treatment decision, and the first intravenous methylprednisolone dose marked the onset of exposure. The first evaluable scan obtained after END confirmation but before completion of the treatment decision was designated the index scan. All evaluable scans obtained after the index time through 72 hours after EVT were reviewed. For patients who met the primary composite outcome, the endpoint scan was the first scan showing a new abnormality or progression of a preexisting abnormality that met the specified progression criteria; for patients who did not meet the primary outcome, it was the last evaluable scan within 72 hours after EVT. Multivariate logistic regression, propensity-score analyses, a restricted analysis among patients with modified Thrombolysis in Cerebral Infarction (mTICI) grade 2b–3, and false discovery rate (FDR) correction were used.

**Results:**

The composite outcome occurred in 42 of 96 treated patients and 122 of 201 untreated patients (43.8% vs 60.7%; *p* = 0.006). After multivariate adjustment, short-course methylprednisolone was associated with lower odds of the composite outcome (adjusted odds ratio, 0.46; 95% confidence interval [CI], 0.27–0.80; *p* = 0.005). The results were directionally consistent among patients with mTICI grades 2b–3 (adjusted odds ratio, 0.48; 95% CI, 0.27–0.86; *p* = 0.013), whereas the estimate for mTICI grades 0–2a was imprecise (*p* = 0.699). The index-to-endpoint-scan interval, endpoint imaging modality, and post-index scan frequency were comparable between groups. After FDR correction across 17 exploratory outcomes, only new or worsening cerebral edema remained statistically significant (*q* = 0.034). Neither the 90-day 3-category modified Rankin Scale (mRS) distribution nor the comparison of mRS 0–2 remained statistically significant after correction. Safety estimates were imprecise.

**Conclusions:**

Short-course methylprednisolone was associated with lower odds of the study-defined early post-EVT composite imaging abnormality, primarily reflecting edema-related findings. This observational association did not establish functional benefit and requires prospective confirmation.

## 1. Introduction

Acute ischemic stroke is a leading cause of death and long-term disability worldwide, and strokes caused by large-vessel occlusion are generally associated with more severe neurological deficits and a greater burden of disability [1]. Current reperfusion guidelines recommend endovascular thrombectomy (EVT) as a cornerstone treatment for eligible patients with large-vessel occlusion [2]. Recent evidence in patients with large ischemic cores has further expanded the applicability of EVT while underscoring the need to monitor postprocedural tissue injury and complications [3].

Successful recanalization of a large vessel does not necessarily ensure adequate microcirculatory tissue perfusion; distal hypoperfusion or no-reflow may continue to limit tissue recovery [4]. Early neurological deterioration (END), typically manifested by an increase in National Institutes of Health Stroke Scale (NIHSS) score, worsening level of consciousness, or a new focal deficit, is an important clinical phenotype during the early course of stroke [5]. END after EVT is associated with poorer functional outcomes and increased risks of symptomatic intracranial hemorrhage and death; potential mechanisms include reocclusion, infarct progression, cerebral edema, hemorrhagic transformation, microcirculatory dysfunction, and systemic physiological disturbances [6].

Ischemic cerebral edema is driven by both cytotoxic and vasogenic components and can progress to mass effect and midline shift [7]. Blood-brain barrier disruption evolves dynamically and may involve abnormal endothelial transport, tight-junction injury, and inflammatory-cell infiltration [8]. Oxidative stress and inflammatory cascades further amplify injury to the neurovascular unit, providing a biological rationale for early anti-inflammatory and barrier-modulating interventions [9]. Neuroprotective strategies in the reperfusion era must account for treatment timing, target population, mechanistic specificity, and clinical outcomes [10]. Methylprednisolone has rapid anti-inflammatory effects and can modulate vascular permeability; however, its association with early imaging findings in patients who develop END after EVT remains unclear. We assessed the association between short-course methylprednisolone and the study-defined early post-EVT composite imaging abnormality after the index scan and evaluated the robustness of the findings using multivariable adjustment, propensity-score methods, and analyses stratified by modified Thrombolysis in Cerebral Infarction (mTICI) grade.

## 2. Materials and Methods

### 2.1 Study Design and Patients

This was a single-center retrospective analysis. We consecutively collected clinical, laboratory, endovascular treatment, and imaging data for patients who underwent EVT for acute ischemic stroke at an advanced stroke center with an annual EVT volume of approximately 200 procedures between January 1, 2020, and December 31, 2023, and developed END within 72 hours after EVT. Acute ischemic stroke was diagnosed on the basis of clinical presentation and cranial computed tomography (CT) or magnetic resonance imaging (MRI); occlusion of the culprit vessel was confirmed by CT angiography, magnetic resonance angiography, or digital subtraction angiography. This study was reported in accordance with the STROBE statement (**Supplementary Material**).

Eligibility criteria were as follows: age ≥18 years; diagnosis of acute ischemic stroke; receipt of EVT; development of END within 72 hours after EVT; availability of an evaluable cranial CT or MRI scan obtained after END confirmation and before completion of the treatment decision; and availability of preprocedural baseline data, periprocedural treatment records, the index scan, imaging follow-up within 72 hours after EVT, and key clinical and safety outcome data.

Exclusion criteria were as follows: intracranial hemorrhage on preprocedural imaging; intracranial tumor, severe traumatic brain injury, intracranial infection, or another condition that interfered with imaging interpretation; prestroke modified Rankin Scale (mRS) score >2; postprocedural neurological deterioration attributable to reocclusion, recurrent stroke, status epilepticus, severe hypoglycemia, shock, severe infection, or another clearly defined cause; long-term or high-dose systemic glucocorticoid therapy before or after EVT for another condition; missing index or 72-hour follow-up imaging or inadequate image quality; or missing key clinical, laboratory, or NIHSS data.

### 2.2 Definition of Early Neurological Deterioration

END was defined by any of the following within 72 hours after EVT: an increase of ≥4 points from the immediately postprocedural NIHSS score or the lowest NIHSS score within 24 hours after EVT; an increase of ≥2 points on the level-of-consciousness item; or a new focal neurological deficit judged by a neurologist to reflect progression of the lesion in the culprit vascular territory [11]. These criteria could overlap. In sedated or intubated patients and those with metabolic disturbances, END was adjudicated using the first assessable time point after awakening, serial neurological examinations, and contemporaneous imaging.

### 2.3 Index Time, Treatment Exposure, and Grouping

The index time was defined as completion of the post-END treatment decision and served as the common time zero for both groups. The index scan was the first evaluable CT or MRI examination obtained after END confirmation and before the index time. In the methylprednisolone group, patients received intravenous methylprednisolone sodium succinate (Solu-Medrol®, 40 mg/vial; approval No. H20170197; Pfizer Manufacturing Belgium NV, Puurs-Sint-Amands, Belgium), and the first intravenous dose marked the onset of exposure; the index scan was required to precede the first dose. All evaluable scans obtained after the index time through 72 hours after EVT were reviewed. For patients who met the primary composite outcome, the endpoint scan was the first scan showing a new abnormality or progression of a preexisting abnormality that met the specified progression criteria; for patients who did not meet the primary outcome, the endpoint scan was the last evaluable scan within 72 hours after EVT. In the methylprednisolone group, the endpoint scan was required to occur after the first dose; in the no-methylprednisolone group, it was required to occur after the index time. All time intervals, the number of evaluable post-index scans, the number of doses administered before the endpoint scan, and the cumulative dose administered before the endpoint scan were calculated at the patient level from timestamps and actual administration records. Short-course methylprednisolone was defined as intravenous treatment initiated within 24 hours after END and continued for ≤5 days. Patients who received neither methylprednisolone nor another systemic glucocorticoid were assigned to the no-methylprednisolone group.

During the study period, no standardized methylprednisolone treatment pathway was implemented, and no fixed NIHSS, Alberta Stroke Program Early Computed Tomography Score (ASPECTS), or imaging threshold was specified. Treatment was selected by the attending stroke team on the basis of serial neurological changes, index imaging, anticipated benefit, and relative contraindications. Potential decision factors recorded in routine medical records included persistent or progressive END, cerebral edema or mass effect, contrast-related changes, infarct extent, collateral status, active infection, uncontrolled hyperglycemia, and gastrointestinal bleeding; documentation completeness varied among patients, and no standardized structured template was used. Statistical adjustment was limited to variables measured before the index time with complete documentation.

All patients received comprehensive acute ischemic stroke care, including management of blood pressure, glucose, temperature, and intracranial pressure; nutritional support; and prevention and treatment of complications. Intravenous thrombolysis, antiplatelet therapy, anticoagulation, lipid-lowering therapy, and other supportive treatments were determined by the treating team according to the clinical condition, index imaging, and bleeding risk. Any pre-index antithrombotic therapy was defined as antiplatelet or anticoagulant therapy initiated before the index time.

### 2.4 Endovascular Treatment and Periprocedural Data

Eligibility for EVT was determined according to symptom-onset time, baseline neurological deficit, imaging findings, and clinical practice. EVT strategies included stent-retriever thrombectomy, direct aspiration, or a combined technique. We recorded occlusion site, anesthesia type, bridging intravenous thrombolysis, thrombectomy strategy, onset-to-admission time, onset-to-puncture time, puncture-to-final-angiography time, onset-to-final-angiography time, number of thrombectomy passes, rescue treatment, and final mTICI grade; mTICI grade 2b–3 was defined as successful reperfusion [12]. END onset time was defined as the interval from completion of final angiography to the first time the END criteria were met, and thrombectomy-to-index time as the interval from completion of final angiography to the index time. Puncture-to-reperfusion and onset-to-reperfusion times were calculated only for patients with mTICI grade 2b–3. Pre-index blood pressure and glucose variables included mean systolic blood pressure, peak systolic blood pressure, 24-hour postprocedural systolic blood pressure variability, and peak glucose. Systolic blood pressure variability was defined as the standard deviation of all systolic blood pressure measurements obtained within 24 hours after EVT.

### 2.5 Imaging Assessment and Outcome Definitions

We collected preprocedural CT or MRI, CT angiography or magnetic resonance angiography, intraprocedural digital subtraction angiography, the index scan, and follow-up CT or MRI obtained within 72 hours after EVT. Preprocedural imaging assessments included occlusion site, ASPECTS, collateral status, and early ischemic changes. The index scan was assessed for mild CT hyperdensity or a corresponding MRI signal abnormality, mild hemorrhagic transformation, mild cerebral edema, and any mild related abnormality present before the treatment decision; only abnormalities that newly appeared after the index scan or progressed to the specified progression criteria were counted as outcomes.

The primary imaging outcome was the study-defined early post-EVT composite imaging abnormality, defined as a new or progressive abnormality after the index scan and within 72 hours after EVT. Hemorrhagic transformation was classified according to the European Cooperative Acute Stroke Study (ECASS) imaging phenotypes as hemorrhagic infarction type 1 (HI1), hemorrhagic infarction type 2 (HI2), parenchymal hematoma type 1 (PH1), or parenchymal hematoma type 2 (PH2) [13]. The composite outcome also included CT hyperdensity or a corresponding blood- or contrast-related MRI signal abnormality, new or worsening cerebral edema, abnormal enhancement or exudative changes in the infarcted region, and progression of mass effect. Because contrast-related hyperdensity and early hemorrhage can overlap on conventional CT, adjudication incorporated serial imaging, MRI sequences, or changes on subsequent CT [14]. Cerebral edema was identified on the basis of sulcal effacement, ventricular compression, reduced gray-white matter differentiation, midline shift, and associated mass effect [15]. This composite outcome was an operational imaging phenotype; the composite and each imaging component were reported separately. Escalation of intracranial pressure therapy or decompressive craniectomy was reported separately as an imaging-related management outcome and was not a component of the composite endpoint. In the methylprednisolone group, a relevant abnormality had to first meet the criteria for a new or progressive abnormality on imaging obtained after the first dose; its onset was interval-censored between the index scan and the first positive scan.

Patients with mTICI grade 2b–3 underwent a clinically more specific restricted analysis, whereas patients with mTICI grade 0–2a were analyzed separately in an exploratory analysis. In the restricted multivariable model, onset-to-reperfusion time replaced onset-to-final-angiography time, and the mTICI variable, which was constant within the subgroup, was removed. All images were independently reviewed by 2 physicians blinded to treatment group and clinical outcomes; disagreements were adjudicated by a third senior neuroradiologist. Using the original paired ratings before adjudication, Cohen *κ* was calculated for binary outcomes and quadratically weighted *κ* for ordered ECASS subtypes. Among patients whose endpoint scan was CT, unweighted Cohen *κ* was calculated for the nominal 3-category classification of no relevant hyperdensity, contrast-related hyperdensity, or hemorrhagic change. The 95% confidence intervals for *κ* were estimated using 1000 bootstrap resamples.

### 2.6 Clinical, Functional, and Safety Outcomes

Demographic characteristics, vascular risk factors, prior medications, prestroke mRS score, baseline NIHSS score, stroke etiology, admission vital signs, and periprocedural blood pressure and glucose variables were extracted from the electronic medical record. Laboratory variables included complete blood count, fasting glucose, glycated hemoglobin, lipid profile, coagulation measures, D-dimer, C-reactive protein, albumin, and liver and renal function tests. Neurological status was assessed using the NIHSS [16]. Ninety-day functional outcomes were assessed with the mRS by uniformly trained evaluators during an outpatient visit or by telephone; the 3-category mRS distribution (scores 0–2, 3–4, and 5–6) was the principal functional description, and mRS 0–2 was an exploratory dichotomous measure [17]. Safety outcomes included pneumonia, gastrointestinal bleeding, hyperglycemic events, electrolyte disturbances, and other adverse events potentially related to glucocorticoids. A hyperglycemic event was operationally defined as a random glucose level ≥10.0 mmol/L during hospitalization or the need to initiate insulin, and was interpreted in the context of clinical features of glucocorticoid-induced hyperglycemia [18].

### 2.7 Statistical Analysis

Statistical analyses were performed using R version 4.3.2 (R Foundation for Statistical Computing, Vienna, Austria) and Statistical Package for the Social Sciences (SPSS) version 26.0 (IBM Corp., Armonk, NY, USA). Continuous variables were summarized as mean ± standard deviation or median [interquartile range], as appropriate. Normally distributed variables were compared using the independent-samples *t* test, and nonnormally distributed variables using the Mann–Whitney *U* test; categorical variables were compared using the Pearson *χ^2^* test, Fisher exact test, or Fisher–Freeman–Halton exact test. The primary association was estimated using multivariable logistic regression and expressed as an odds ratio (OR) with a 95% confidence interval (CI); robustness was evaluated using propensity-score matching, stabilized inverse probability weighting, and propensity-score covariate adjustment. Adjustment covariates were selected on the basis of clinical relevance, temporal ordering, and prior evidence and were retained in the model. The propensity-score model included only pre-index variables. One-to-one nearest-neighbor matching was performed with a caliper width equal to 0.20 standard deviations of the logit of the propensity score; conditional logistic regression and the exact McNemar test were used after matching. Stabilized weights were truncated at the 1st and 99th percentiles, and robust standard errors were used. An absolute standardized difference <0.10 indicated adequate covariate balance.

In the mTICI grade 2b–3 restricted model, onset-to-reperfusion time replaced onset-to-final-angiography time, and the mTICI variable, which was constant within the subgroup, was removed. The sole primary imaging outcome was tested using a 2-sided *α* = 0.05. Seven secondary imaging outcomes, 1 imaging-related management outcome, 4 clinical or functional outcomes, and 5 safety outcomes (17 total) were treated as a single exploratory testing family and controlled using the Benjamini–Hochberg false discovery rate (FDR) procedure. Both raw *p* values and *q* values were reported, with *q* < 0.05 indicating statistical significance after correction. Baseline comparisons were descriptive and were not included in the FDR family. Imaging agreement was summarized using *κ* with 95% CIs. The multivariable model was used for confounding adjustment and to characterize internal performance; it included 164 events and 15 parameters. We reported the apparent area under the receiver operating characteristic curve (AUC), the optimism-corrected AUC from 1000 bootstrap resamples, the AUC from 10 repeats of 10-fold cross-validation, the optimism-corrected calibration intercept and slope, apparent and optimism-corrected Brier scores, and exploratory subgroup AUCs. Absolute risk differences with 95% CIs for safety outcomes were calculated using the Newcombe method.

No variables in the primary model were missing. Glycated hemoglobin, D-dimer, and C-reactive protein had only small amounts of missing data and were used solely in descriptive analyses; comparisons were based on the available nonmissing observations, without imputation. Multicollinearity was assessed using variance inflation factors. All tests were 2-sided.

## 3. Results

### 3.1 Patients

We screened 354 patients with acute ischemic stroke who developed END within 72 hours after EVT. We excluded patients whose neurological deterioration was clearly attributable to reocclusion, recurrent stroke, status epilepticus, severe infection, shock, or metabolic disturbance; those with missing key imaging or clinical data, prestroke mRS score >2, or systemic glucocorticoid use for another condition; and those meeting other exclusion criteria. The final cohort comprised 297 patients, including 96 in the methylprednisolone group and 201 in the no-methylprednisolone group. All patients completed the 90-day mRS assessment.

### 3.2 Baseline Clinical Characteristics

No statistically significant between-group differences were observed in age, sex, prestroke mRS distribution, hypertension, diabetes, atrial fibrillation, coronary artery disease, prior stroke or transient ischemic attack, dyslipidemia, current smoking, prior antiplatelet therapy, prior anticoagulation, prior statin therapy, admission systolic blood pressure, admission diastolic blood pressure, heart rate, temperature, or stroke etiology (all *p* > 0.05). The baseline NIHSS score was numerically higher in the methylprednisolone group, although the difference was not statistically significant (*p* = 0.082); this pattern suggested that treatment selection may have been related to disease severity (Table 1).

**Table 1.** **Baseline clinical characteristics by treatment group**.

Variable		Methylprednisolone group (*n* = 96)	No-methylprednisolone group (*n* = 201)	Statistic or test	*p* value
Age, y		69.5 ± 10.8	68.8 ± 11.5	*t* = 0.51	0.610
Male sex		56 (58.3)	119 (59.2)	*χ^2^* = 0.02	0.887
Prestroke modified Rankin Scale (mRS) score distribution				*χ^2^* = 0.12	0.940
	Score 0	55 (57.3)	119 (59.2)	—	—
	Score 1	32 (33.3)	65 (32.3)	—	—
	Score 2	9 (9.4)	17 (8.5)	—	—
Hypertension		68 (70.8)	132 (65.7)	*χ^2^* = 0.79	0.375
Diabetes mellitus		30 (31.2)	52 (25.9)	*χ^2^* = 0.94	0.332
Atrial fibrillation		45 (46.9)	85 (42.3)	*χ^2^* = 0.56	0.456
Coronary artery disease		20 (20.8)	36 (17.9)	*χ^2^* = 0.36	0.547
Prior stroke or transient ischemic attack		21 (21.9)	42 (20.9)	*χ^2^* = 0.04	0.847
Dyslipidemia		37 (38.5)	76 (37.8)	*χ^2^* = 0.01	0.903
Current smoking		31 (32.3)	67 (33.3)	*χ^2^* = 0.03	0.858
Prior antiplatelet therapy		25 (26.0)	50 (24.9)	*χ^2^* = 0.05	0.829
Prior anticoagulation		15 (15.6)	29 (14.4)	*χ^2^* = 0.07	0.786
Prior statin therapy		29 (30.2)	57 (28.4)	*χ^2^* = 0.11	0.742
Baseline National Institutes of Health Stroke Scale (NIHSS) score		18 [14–22]	17 [13–21]	*Z* = –1.74	0.082
Admission systolic blood pressure, mmHg		155 ± 24	153 ± 23	*t* = 0.68	0.497
Admission diastolic blood pressure, mmHg		86 ± 15	85 ± 14	*t* = 0.55	0.584
Admission heart rate, beats/min		82 ± 17	81 ± 16	*t* = 0.48	0.630
Admission temperature, °C		36.7 ± 0.4	36.7 ± 0.4	*t* = 0.00	1.000
Stroke etiology				Fisher–Freeman–Halton exact test	0.922
	Large-artery atherosclerosis	36 (37.5)	71 (35.3)	—	—
	Cardioembolism	43 (44.8)	90 (44.8)	—	—
	Other determined etiology	5 (5.2)	9 (4.5)	—	—
	Undetermined etiology	12 (12.5)	31 (15.4)	—	—

Note: Normally distributed continuous variables are presented as mean ± standard deviation, nonnormally distributed continuous variables as median [interquartile range], and categorical variables as number (%). *n* indicates sample size; *t*, the independent-samples *t* test statistic; *Z*, the standardized Mann–Whitney *U* test statistic; *χ^2^*, the Pearson chi-square statistic; and *p*, the 2-sided *p* value. The Fisher–Freeman–Halton exact test was used for multicategory contingency tables. An em dash indicates not applicable or not separately tested. mRS, modified Rankin Scale; NIHSS, National Institutes of Health Stroke Scale.

### 3.3 Admission Laboratory Variables

White blood cell count, neutrophil percentage, fasting glucose, glycated hemoglobin, total cholesterol, triglycerides, low-density lipoprotein cholesterol, international normalized ratio, activated partial thromboplastin time, fibrinogen, D-dimer, C-reactive protein, alanine aminotransferase, aspartate aminotransferase, and creatinine were numerically higher in the methylprednisolone group, whereas hemoglobin, platelet count, high-density lipoprotein cholesterol, albumin, and estimated glomerular filtration rate were numerically lower. None of these between-group differences was statistically significant (all *p* > 0.05). Data were missing for glycated hemoglobin in 9 patients, D-dimer in 8, and C-reactive protein in 10; comparisons used the available nonmissing observations (**Supplementary Table 1**).

### 3.4 Preprocedural Imaging, Endovascular Treatment, and Periprocedural Data

The methylprednisolone group had numerically lower baseline ASPECTS, higher proportions of preprocedural early ischemic changes and poor collateral status, longer onset-to-puncture, puncture-to-final-angiography, onset-to-final-angiography, puncture-to-reperfusion, and onset-to-reperfusion times among patients with mTICI grade 2b–3, and higher pre-index mean and peak systolic blood pressure, 24-hour postprocedural systolic blood pressure variability, and peak glucose; none of these differences was statistically significant. Baseline imaging modality, occlusion site, bridging intravenous thrombolysis, anesthesia type, thrombectomy strategy, onset-to-admission time, number of thrombectomy passes, intraprocedural rescue treatment, mTICI distribution, proportion with mTICI grade 2b–3, postprocedural antiplatelet therapy, postprocedural anticoagulation, postprocedural statin therapy, and any pre-index antithrombotic therapy also did not differ significantly between groups (all *p* > 0.05) (Table 2).

**Table 2.** **Preprocedural imaging, endovascular treatment, and periprocedural data**.

Variable		Methylprednisolone group (*n* = 96)	No-methylprednisolone group (*n* = 201)	Statistic	*p* value
Baseline imaging modality				*χ^2^* = 0.15	0.698
	Computed tomography (CT)	80 (83.3)	171 (85.1)	—	—
	Magnetic resonance imaging (MRI)	16 (16.7)	30 (14.9)	—	—
Baseline Alberta Stroke Program Early Computed Tomography Score (ASPECTS)		8 [6–9]	8 [7–9]	*Z* = –1.86	0.063
Preprocedural early ischemic changes		57 (59.4)	105 (52.2)	*χ^2^* = 1.33	0.248
Collateral status				*χ^2^* = 2.60	0.272
	Good	29 (30.2)	77 (38.3)	—	—
	Intermediate	26 (27.1)	56 (27.9)	—	—
	Poor	41 (42.7)	68 (33.8)	—	—
Occlusion site				*χ^2^* = 0.63	0.960
	Internal carotid artery terminus	25 (26.0)	46 (22.9)	—	—
	Middle cerebral artery M1 segment	43 (44.8)	96 (47.8)	—	—
	Middle cerebral artery M2 segment	9 (9.4)	16 (8.0)	—	—
	Basilar artery	11 (11.5)	25 (12.4)	—	—
	Tandem lesion	8 (8.3)	18 (9.0)	—	—
Bridging intravenous thrombolysis		32 (33.3)	62 (30.8)	*χ^2^* = 0.19	0.666
General anesthesia		43 (44.8)	91 (45.3)	*χ^2^* = 0.01	0.938
Thrombectomy strategy				*χ^2^* = 0.10	0.949
	Stent-retriever thrombectomy	33 (34.4)	68 (33.8)	—	—
	Direct aspiration	26 (27.1)	58 (28.9)	—	—
	Combined technique	37 (38.5)	75 (37.3)	—	—
Onset-to-admission time, min		145 [85–240]	150 [90–245]	*Z* = –0.36	0.719
Onset-to-puncture time, min		292 [210–420]	280 [205–390]	*Z* = –0.79	0.430
Puncture-to-final-angiography time, min		58 [42–86]	56 [40–83]	*Z* = –0.66	0.509
Onset-to-final-angiography time, min		366 [286–505]	352 [279–486]	*Z* = –0.77	0.441
Puncture-to-reperfusion time in patients with modified Thrombolysis in Cerebral Infarction (mTICI) grade 2b–3, min		52 [36–78]; *n* = 84	49 [34–74]; *n* = 171	*Z* = –0.97	0.332
Onset-to-reperfusion time in patients with mTICI grade 2b–3, min		354 [275–492]; *n* = 84	341 [270–468]; *n* = 171	*Z* = –0.83	0.407
Number of thrombectomy passes		2 [1–3]	2 [1–3]	*Z* = –0.70	0.484
Intraprocedural rescue treatment				*χ^2^* = 0.18	0.980
	None	67 (69.8)	145 (72.1)	—	—
	Intra-arterial thrombolysis	10 (10.4)	19 (9.5)	—	—
	Balloon angioplasty or stent placement	12 (12.5)	23 (11.4)	—	—
	Other rescue measures	7 (7.3)	14 (7.0)	—	—
mTICI grade				*χ^2^* = 1.19	0.553
	Grade 0–2a	12 (12.5)	30 (14.9)	—	—
	Grade 2b	40 (41.7)	92 (45.8)	—	—
	Grade 2c–3	44 (45.8)	79 (39.3)	—	—
Successful reperfusion, mTICI grade 2b–3		84 (87.5)	171 (85.1)	*χ^2^* = 0.31	0.575
Pre-index mean systolic blood pressure, mm Hg		144 ± 15	142 ± 14	*t* = 1.10	0.274
Pre-index peak systolic blood pressure, mm Hg		164 ± 21	160 ± 20	*t* = 1.56	0.121
24-hour postprocedural systolic blood pressure variability, mm Hg		13.8 ± 6.2	13.1 ± 6.0	*t* = 0.92	0.359
Pre-index peak glucose, mmol/L		9.4 [7.4–12.1]	8.8 [7.0–11.4]	*Z* = –1.70	0.089
Postprocedural antiplatelet therapy		71 (74.0)	151 (75.1)	*χ^2^* = 0.05	0.829
Postprocedural anticoagulation		18 (18.8)	40 (19.9)	*χ^2^* = 0.05	0.815
Postprocedural statin therapy		85 (88.5)	179 (89.1)	*χ^2^* = 0.02	0.895
Any pre-index antithrombotic therapy		39 (40.6)	84 (41.8)	*χ^2^* = 0.04	0.849

Note: Data are presented as mean ± standard deviation, median [interquartile range], or number (%). *n* indicates sample size; *t*, the independent-samples *t* test statistic; *Z*, the standardized Mann–Whitney *U* test statistic; *χ^2^*, the Pearson chi-square statistic; and *p*, the 2-sided *p* value. An em dash indicates that no separate test was performed. CT, computed tomography; MRI, magnetic resonance imaging; ASPECTS, Alberta Stroke Program Early Computed Tomography Score; mTICI, modified Thrombolysis in Cerebral Infarction; M1 and M2, the corresponding anatomical segments of the middle cerebral artery. Puncture-to-reperfusion and onset-to-reperfusion times were calculated only in patients with mTICI grade 2b–3.

### 3.5 Neurological and Treatment Characteristics After END

END onset time, thrombectomy-to-index time, END-to-index time, index-to-endpoint-scan time, endpoint imaging modality, number of evaluable post-index scans, proportion with at least 2 post-index scans, increase in NIHSS score, NIHSS score increase ≥4 points, level-of-consciousness item increase ≥2 points, and new focal neurological deficit did not differ significantly between groups (all *p* > 0.05). In the methylprednisolone group, index-to-first-dose time, first-dose-to-endpoint-scan time, number of doses and cumulative dose before the endpoint scan, END-to-first-dose time, treatment duration, daily dose, cumulative course dose, and dosing frequency were summarized from actual administration records. In all treated patients, the index scan preceded the first dose and the endpoint scan followed the first dose. In all 42 treated patients who met the primary outcome, the relevant abnormality first met the outcome criterion on imaging obtained after the first dose; its onset was interval-censored between the index scan and the first positive scan (Table 3).

**Table 3.** **Early neurological deterioration, treatment exposure, and imaging timing**.

Variable		Methylprednisolone group (*n* = 96)	No-methylprednisolone group (*n* = 201)	Statistic	*p* value
Early neurological deterioration (END) onset time, h		11 [6–19]	12 [6–20]	*Z* = –0.45	0.653
Thrombectomy-to-index time, h		16 [11–26]	16 [10–27]	*Z* = –0.22	0.826
END-to-index time, h		4.3 [2.1–8.0]	4.5 [2.2–8.3]	*Z* = –0.43	0.667
Index-to-endpoint-scan time, h		26.5 [17.7–39.1]	27.0 [18.0–39.8]	*Z* = –0.35	0.726
Endpoint imaging modality				*χ^2^* = 0.09	0.766
	Computed tomography (CT)	82 (85.4)	169 (84.1)	—	—
	Magnetic resonance imaging (MRI)	14 (14.6)	32 (15.9)	—	—
Number of evaluable post-index scans		2 [1–2]	2 [1–2]	*Z* = –0.24	0.814
At least 2 post-index scans		57 (59.4)	117 (58.2)	*χ^2^* = 0.04	0.849
Increase in National Institutes of Health Stroke Scale (NIHSS) score		5 [4–7]	5 [4–8]	*Z* = –0.41	0.682
NIHSS score increase ≥4 points		70 (72.9)	152 (75.6)	*χ^2^* = 0.25	0.616
Level-of-consciousness item increase ≥2 points		18 (18.8)	40 (19.9)	*χ^2^* = 0.05	0.815
New focal neurological deficit		8 (8.3)	9 (4.5)	*χ^2^* = 1.79	0.181
Index-to-first-dose time, h		0.8 [0.4–1.4]	Not applicable	—	—
First-dose-to-endpoint-scan time, h		25.6 [16.9–37.8]	Not applicable	—	—
Number of doses before endpoint scan		2 [1–2]	Not applicable	—	—
Cumulative dose before endpoint scan, mg		160 [80–240]	Not applicable	—	—
Index scan before first dose		96 (100.0)	Not applicable	—	—
Endpoint scan after first dose		96 (100.0)	Not applicable	—	—
END-to-first-dose time, h		5.2 [2.8–9.6]	Not applicable	—	—
Treatment duration, d		3 [3–5]	Not applicable	—	—
Daily dose, mg		80 [80–120]	Not applicable	—	—
Cumulative course dose, mg		320 [240–480]	Not applicable	—	—
Once-daily dosing		74 (77.1)	Not applicable	—	—
Twice-daily dosing		22 (22.9)	Not applicable	—	—
Primary outcome first met on imaging obtained after the first dose		42/42 (100.0)	Not applicable	—	—

Note: Data are presented as median [interquartile range] or number (%). *Z* indicates the standardized Mann–Whitney *U* test statistic; *χ^2^*, the Pearson chi-square statistic; and *p*, the 2-sided *p* value. An em dash indicates not applicable or not separately tested. END, early neurological deterioration; EVT, endovascular thrombectomy. END onset time and thrombectomy-to-index time were both measured from completion of final angiography. The endpoint scan was the first positive scan in patients who met the primary outcome or the last evaluable scan within 72 hours after EVT in patients who did not. The number of evaluable post-index scans included all CT or MRI examinations obtained after the index time through 72 hours that could be used for outcome determination. END criteria could overlap.

### 3.6 Primary Imaging Outcome and Inter-Reader Agreement

At the index scan, mild CT hyperdensity or corresponding MRI signal abnormality, mild hemorrhagic transformation, mild cerebral edema, and any mild related change were numerically more frequent in the methylprednisolone group, with no statistically significant between-group differences (all *p* > 0.05). The study-defined early post-EVT composite imaging abnormality was less frequent in the methylprednisolone group (*p* = 0.006). The component imaging outcomes—hemorrhagic transformation, parenchymal hematoma, CT hyperdensity or corresponding MRI signal abnormality, new or worsening cerebral edema, abnormal enhancement or exudative change, and midline shift—and the imaging-related management outcome of escalation of intracranial pressure therapy or decompressive craniectomy were also numerically less frequent in the methylprednisolone group. After FDR correction across the 17 exploratory outcomes, only new or worsening cerebral edema remained statistically significant (*p* = 0.002, *q* = 0.034); the ECASS subtype distribution and all other imaging component and management outcomes did not meet *q* < 0.05 (Table 4 and Fig. 1).

**Table 4.** **Index-scan status, primary composite imaging outcome, and components**.

Variable		Methylprednisolone group (*n* = 96)	No-methylprednisolone group (*n* = 201)	Statistic	Raw *p* value	False discovery rate (FDR)-adjusted *q* value
Mild computed tomography (CT) hyperdensity or corresponding magnetic resonance imaging (MRI) signal abnormality on the index scan		18 (18.8)	34 (16.9)	*χ^2^* = 0.15	0.697	—
Mild hemorrhagic transformation on index scan		12 (12.5)	21 (10.4)	*χ^2^* = 0.28	0.599	—
Mild cerebral edema on index scan		22 (22.9)	39 (19.4)	*χ^2^* = 0.49	0.483	—
Any mild related abnormality on index scan		30 (31.2)	54 (26.9)	*χ^2^* = 0.62	0.433	—
Study-defined early post-endovascular thrombectomy (EVT) composite imaging abnormality (primary outcome)		42 (43.8)	122 (60.7)	*χ^2^* = 7.55	0.006	—
Hemorrhagic transformation		37 (38.5)	86 (42.8)	*χ^2^* = 0.48	0.487	0.594
European Cooperative Acute Stroke Study (ECASS) subtype distribution				*χ^2^* = 0.69	0.953	0.953
	No hemorrhagic transformation	59 (61.5)	115 (57.2)	—	—	—
	Hemorrhagic infarction type 1 (HI1)	12 (12.5)	26 (12.9)	—	—	—
	Hemorrhagic infarction type 2 (HI2)	12 (12.5)	26 (12.9)	—	—	—
	Parenchymal hematoma type 1 (PH1)	8 (8.3)	21 (10.4)	—	—	—
	Parenchymal hematoma type 2 (PH2)	5 (5.2)	13 (6.5)	—	—	—
Parenchymal hematoma (PH1–PH2)		13 (13.5)	34 (16.9)	*χ^2^* = 0.56	0.456	0.594
CT hyperdensity or a corresponding MRI signal abnormality		21 (21.9)	60 (29.9)	*χ^2^* = 2.08	0.149	0.253
New or worsening cerebral edema		27 (28.1)	95 (47.3)	*χ^2^* = 9.83	0.002	0.034
Abnormal enhancement or exudative change		16 (16.7)	52 (25.9)	*χ^2^* = 3.12	0.077	0.159
Midline shift		7 (7.3)	34 (16.9)	*χ^2^* = 5.06	0.025	0.139
Imaging-related management outcome: escalation of intracranial pressure therapy or decompressive craniectomy		6 (6.2)	31 (15.4)	*χ^2^* = 5.01	0.025	0.139

Note: Data are presented as number (%). *χ^2^* indicates the Pearson chi-square statistic; *p*, the 2-sided raw *p* value; FDR, false discovery rate; and *q*, the Benjamini–Hochberg-adjusted *p* value. The Fisher–Freeman–Halton exact test was used for multicategory contingency tables. An em dash indicates not applicable or not separately tested. ECASS, European Cooperative Acute Stroke Study; HI1 and HI2, hemorrhagic infarction types 1 and 2; PH1 and PH2, parenchymal hematoma types 1 and 2. The primary outcome was the sole primary test and was not subjected to FDR correction. Seven secondary imaging outcomes and 1 imaging-related management outcome were included with the other 9 clinical, functional, and safety outcomes in the 17-test Benjamini–Hochberg FDR family. Imaging findings could overlap. Escalation of intracranial pressure therapy or decompressive craniectomy was a management outcome. The individual imaging components may overlap and should not be summed to derive the composite endpoint.

**Fig. 1.**
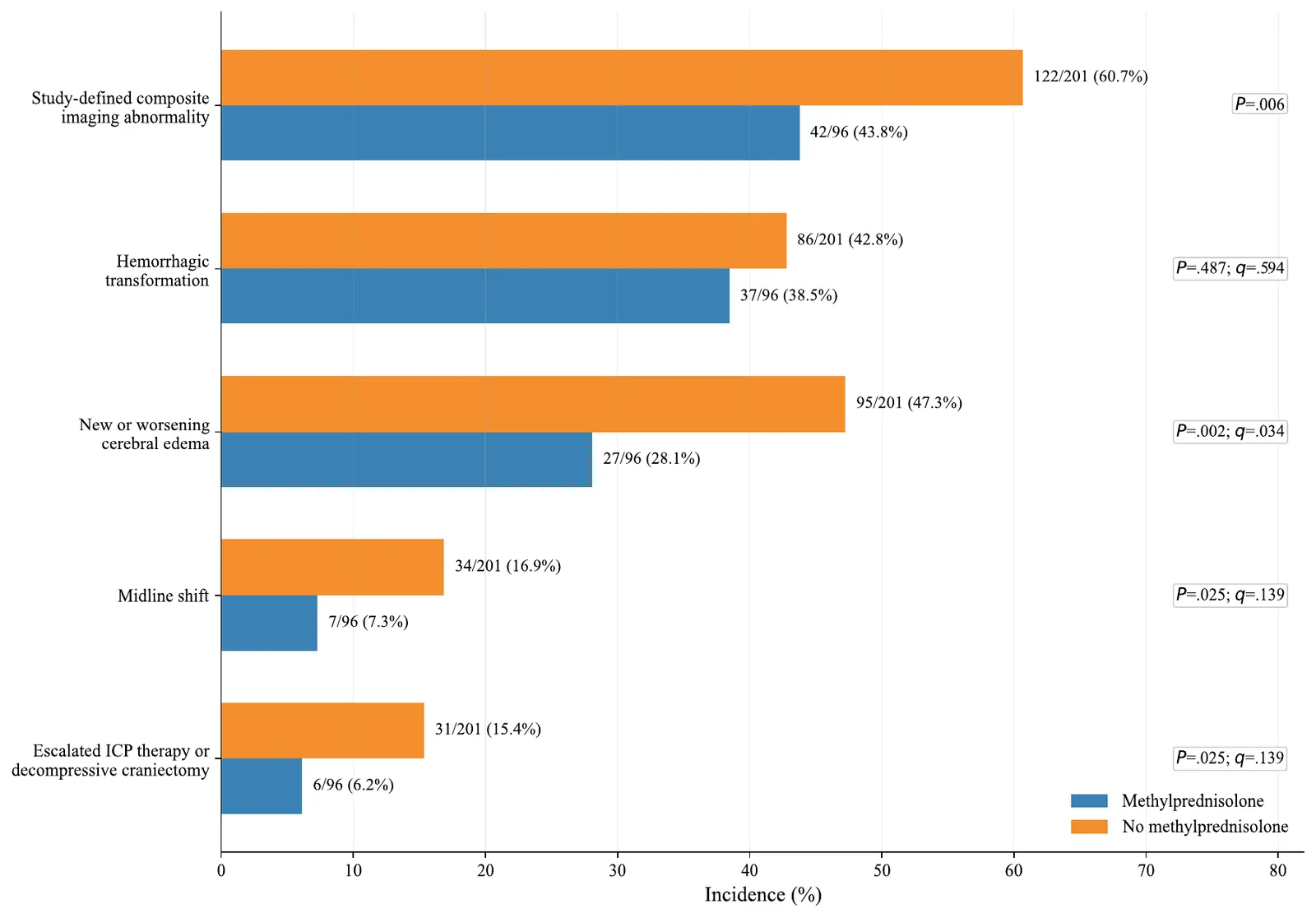
**Incidence of the study-defined early composite imaging abnormality after endovascular thrombectomy (EVT), selected imaging components, and an imaging-related management outcome in the methylprednisolone and no-methylprednisolone groups**. Bar heights show the incidence in each group; labels at the bar ends show the number of events/total number and percentage. Two-sided raw *p* values are shown. For the 4 exploratory outcomes, *q* values after Benjamini–Hochberg false discovery rate (FDR) correction across the 17-test family are also shown. The primary composite outcome was the sole primary test and is reported with a *p* value only. Escalation of intracranial pressure (ICP) therapy or decompressive craniectomy was an imaging-related management outcome.

Before adjudication, the 2 readers showed high agreement for the primary composite outcome, hemorrhagic transformation, ECASS subtype, CT hyperdensity or corresponding MRI signal abnormality, new or worsening cerebral edema, abnormal enhancement or exudative change, and midline shift. Among the 251 patients whose endpoint scan was CT, agreement for the 3-category classification of no relevant hyperdensity, contrast-related hyperdensity, or hemorrhagic change was 92.8%, with an unweighted Cohen *κ* of 0.84 (95% CI, 0.76–0.91); there were 5 direct disagreements between contrast-related hyperdensity and hemorrhagic change (**Supplementary Table 2**).

### 3.7 Neurological, Functional, and Safety Outcomes

The discharge NIHSS score was numerically lower in the methylprednisolone group. The unadjusted comparison was nominally significant, but it was not statistically significant after FDR correction (*p* = 0.033, *q* = 0.139). The 90-day mRS score was numerically lower, the proportion with mRS 0–2 was numerically higher, and the proportion with mRS 5–6 was numerically lower in the methylprednisolone group; however, the mRS score, 3-category distribution, and mRS 0–2 showed no statistically significant between-group differences after FDR correction (all *q* ≥0.139). Pneumonia, gastrointestinal bleeding, hyperglycemic events, electrolyte disturbances, and other related adverse events were numerically more frequent in the methylprednisolone group, but all raw *p* values and *q* values exceeded 0.05. The risk difference for hyperglycemia was positive, and all 95% CIs for safety risk differences were wide (Table 5).

**Table 5.** **Neurological, functional, and safety outcomes**.

Variable		Methylprednisolone group (*n* = 96)	No-methylprednisolone group (*n* = 201)	Statistic/effect estimate	Raw *p* value	False discovery rate (FDR)-adjusted *q* value
Discharge National Institutes of Health Stroke Scale (NIHSS) score		10 [6–15]	12 [7–17]	*Z* = –2.13	0.033	0.139
90-day modified Rankin Scale (mRS) score		4 [2–5]	4 [3–5]	*Z* = –1.83	0.067	0.159
90-day 3-category mRS distribution				*χ^2^* = 5.57	0.062	0.159
	Score 0–2	26 (27.1)	34 (16.9)	—	—	—
	Score 3–4	40 (41.7)	81 (40.3)	—	—	—
	Score 5–6	30 (31.2)	86 (42.8)	—	—	—
90-day mRS 0–2		26 (27.1)	34 (16.9)	Odds ratio (OR) 1.82 (1.02–3.26)	0.041	0.139
Pneumonia		28 (29.2)	51 (25.4)	Risk difference (RD) +3.8 (–6.6 to 15.0) percentage points	0.489	0.594
Gastrointestinal bleeding		5 (5.2)	7 (3.5)	Risk difference (RD) +1.7 (–2.9 to 8.4) percentage points	0.533	0.604
Hyperglycemic events		31 (32.3)	46 (22.9)	Risk difference (RD) +9.4 (–1.2 to 20.6) percentage points	0.084	0.159
Electrolyte disturbance		15 (15.6)	22 (10.9)	Risk difference (RD) +4.7 (–3.1 to 14.0) percentage points	0.253	0.391
Other related adverse events		2 (2.1)	3 (1.5)	Risk difference (RD) +0.6 (–2.6 to 5.9) percentage points	0.660	0.701

Note: Data are presented as median [interquartile range] or number (%). *Z* indicates the standardized Mann–Whitney *U* test statistic; *χ^2^*, the Pearson chi-square statistic; OR, odds ratio; RD, absolute risk difference (methylprednisolone minus no methylprednisolone); CI, confidence interval; *p*, the 2-sided raw *p* value; FDR, false discovery rate; and *q*, the Benjamini–Hochberg-adjusted *p* value. An em dash indicates that no separate test was performed. The 95% CIs for safety outcomes were calculated using the Newcombe method. Clinical, functional, and safety outcomes were included in the 17-test Benjamini–Hochberg FDR family.

### 3.8 Multivariable Association Model and Internal Performance

In multivariable analysis, short-course methylprednisolone was associated with lower odds of the primary composite imaging abnormality. Higher baseline NIHSS, lower ASPECTS, poor collateral status, longer onset-to-final-angiography time, higher pre-index peak systolic blood pressure, and higher peak glucose were associated with the primary outcome; age, sex, terminal internal carotid artery or tandem occlusion, bridging intravenous thrombolysis, mTICI grade 2b–3, number of thrombectomy passes, mild related abnormality on the index scan, and pre-index antithrombotic therapy were not independently associated after adjustment. The model included 164 events and 15 parameters, yielding 10.9 events per parameter. The apparent AUC decreased slightly after bootstrap optimism correction and repeated cross-validation. The optimism-corrected calibration intercept was close to 0, the calibration slope was close to 1, and the optimism-corrected Brier score was slightly higher than the apparent value. Exploratory subgroup AUCs were of similar magnitude for mTICI grade 2b–3, mTICI grade 0–2a, anterior-circulation occlusion, and basilar artery occlusion, although intervals were wide for the mTICI grade 0–2a and basilar artery subgroups (**Supplementary Table 3**, Fig. 2, and **Supplementary Table 4**).

**Fig. 2.**
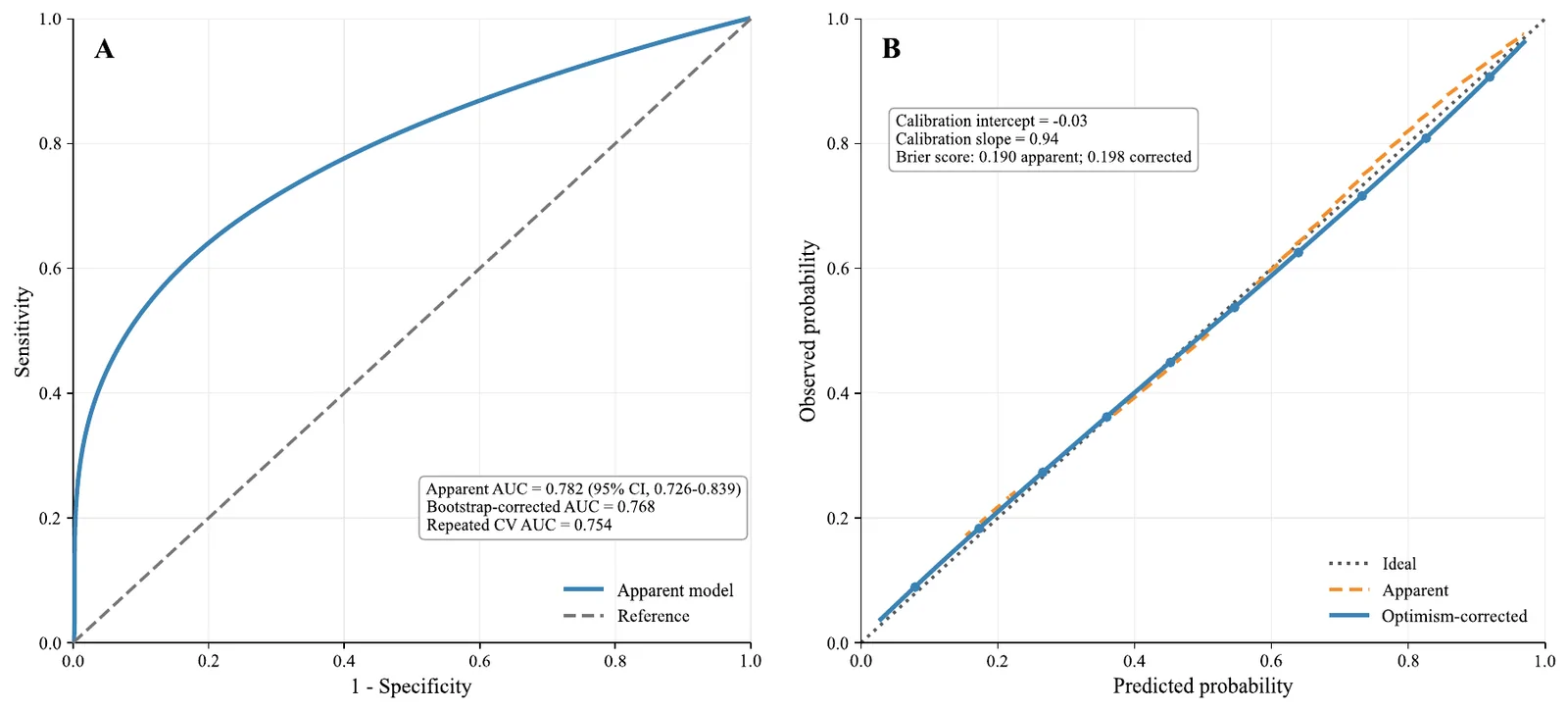
**Internal performance of the multivariable logistic association-adjustment model**. (A) Receiver operating characteristic (ROC) curve: apparent area under the curve (AUC), 0.782 (95% confidence interval [CI], 0.726–0.839); bootstrap optimism-corrected AUC based on 1000 resamples, 0.768; and AUC from 10 repeats of 10-fold cross-validation (CV), 0.754 (percentile interval, 0.716–0.792). (B) Bootstrap calibration plot: the horizontal axis shows model-predicted probability and the vertical axis observed probability; the 45° dotted line denotes ideal calibration, the dashed line the apparent calibration curve, and the solid line the bootstrap optimism-corrected curve. The optimism-corrected calibration intercept was –0.03 and slope 0.94; apparent and optimism-corrected Brier scores were 0.190 and 0.198, respectively. The model included 164 outcome events and 15 parameters.

### 3.9 Propensity-Score and Reperfusion-Status Analyses

All included covariates had absolute standardized differences <0.10 after propensity-score matching. The association was directionally consistent in the unadjusted original cohort, the multivariable-adjusted analysis, propensity-score matching, stabilized inverse probability weighting, and propensity-score covariate adjustment (all *p* < 0.05). The mTICI grade 2b–3 restricted analyses were also directionally consistent (all *p* < 0.05), whereas the composite outcome showed no clear between-group difference in the mTICI grade 0–2a subgroup (*p* = 0.699). Among patients with mTICI grade 0–2a, hemorrhagic transformation, parenchymal hematoma, CT hyperdensity or a corresponding MRI signal abnormality, new or worsening cerebral edema, abnormal enhancement or exudative change, midline shift, and the imaging-related management outcome of escalation of intracranial pressure therapy or decompressive craniectomy were all numerically less frequent in the methylprednisolone group. Given the limited sample size, only descriptive distributions are reported for this subgroup (Fig. 3, Table 6, and **Supplementary Table 5**).

**Fig. 3.**
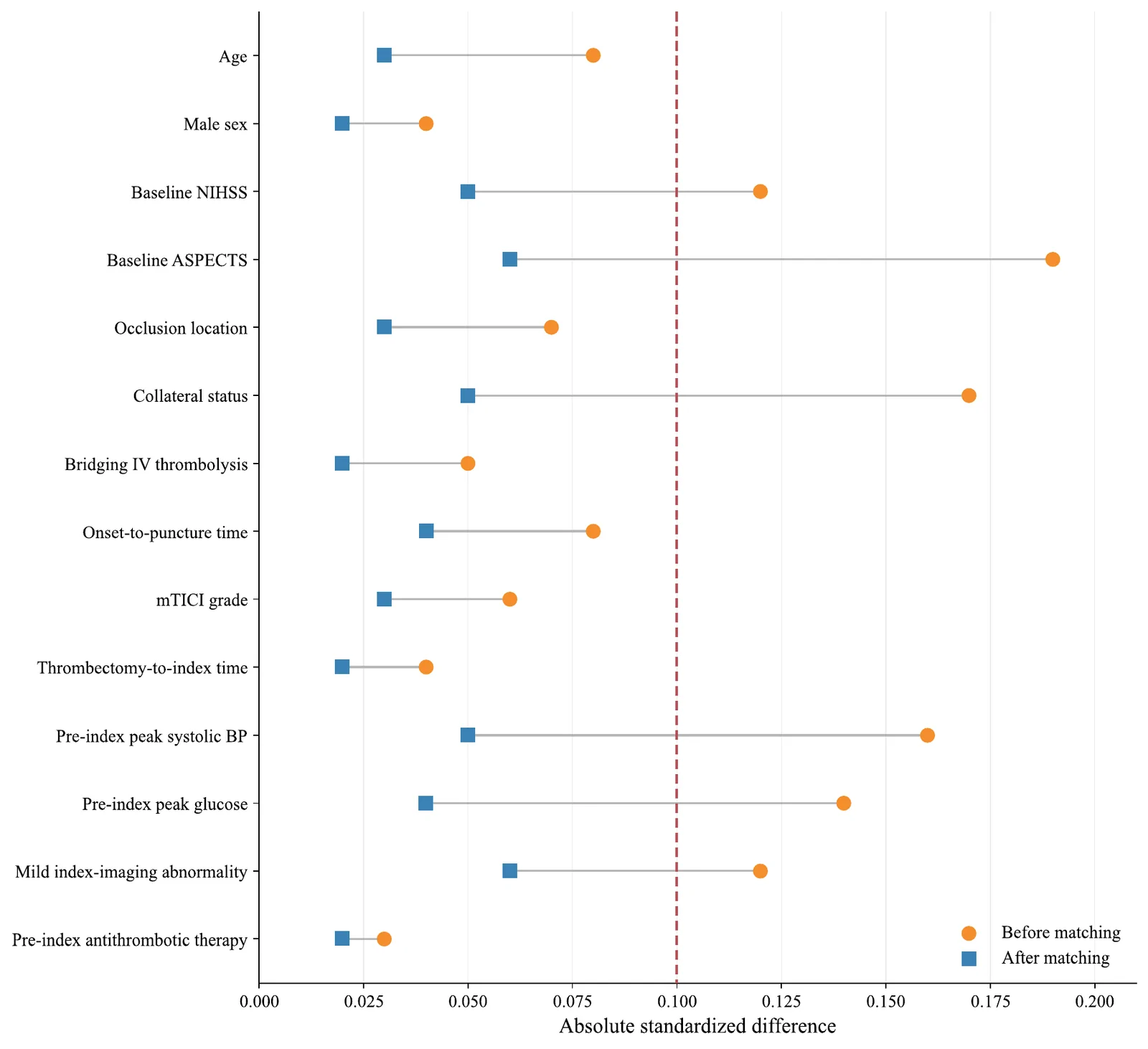
**Covariate balance before and after propensity-score matching**. The Love plot shows absolute standardized differences for age, sex, baseline National Institutes of Health Stroke Scale (NIHSS) score, baseline Alberta Stroke Program Early Computed Tomography Score (ASPECTS), occlusion location, collateral status, bridging intravenous (IV) thrombolysis, onset-to-puncture time, modified Thrombolysis in Cerebral Infarction (mTICI) grade, thrombectomy-to-index time, pre-index peak systolic blood pressure (BP), peak glucose, mild related abnormality on index imaging, and pre-index antithrombotic therapy. Circles indicate values before matching and squares values after matching; the vertical dashed line indicates the 0.10 threshold. ICA, internal carotid artery.

**Table 6.** **Primary association, propensity-score, and analyses stratified by modified thrombolysis in cerebral infarction (mTICI) grade**.

Analysis	Sample	Methylprednisolone group	No-methylprednisolone group	Effect estimate (95% confidence interval [CI])	*p* value
Unadjusted analysis in original cohort	297 patients	42/96 (43.8)	122/201 (60.7)	Odds ratio (OR) 0.50 (0.31–0.82)	0.006
Multivariable-adjusted full-sample analysis	297 patients	—	—	OR 0.46 (0.27–0.80)	0.005
Unadjusted restricted analysis, modified Thrombolysis in Cerebral Infarction (mTICI) grade 2b–3	255 patients	34/84 (40.5)	99/171 (57.9)	OR 0.49 (0.29–0.84)	0.009
Multivariable restricted analysis, mTICI grade 2b–3	255 patients	—	—	OR 0.48 (0.27–0.86)	0.013
Separate exploratory analysis, mTICI grade 0–2a	42 patients	8/12 (66.7)	23/30 (76.7)	OR 0.61 (0.14–2.64)	0.699
1:1 propensity-score matching	172 patients, 86 pairs	36/86 (41.9)	51/86 (59.3)	Matched OR 0.48 (0.24–0.94)	0.032
Stabilized inverse probability weighting	297 patients	Weighted 44.2%	Weighted 59.1%	OR 0.52 (0.32–0.86)	0.010
Propensity-score covariate adjustment	297 patients	—	—	OR 0.47 (0.28–0.80)	0.005

Note: Data are presented as number (%) or weighted percentage. An em dash indicates that group-specific event counts were not reported directly for that analysis. The propensity-score-matched analysis was based on 14:29 discordant pairs; the matched OR and exact 95% CI were calculated from the discordant pairs, and the exact McNemar *p* value was 0.032. The mTICI grade 2b–3 analysis was restricted, whereas the mTICI grade 0–2a analysis was exploratory.

## 4. Discussion

This study included patients with acute ischemic stroke who developed END within 72 hours after EVT. Short-course methylprednisolone was associated with lower odds of the study-defined early post-EVT composite imaging abnormality, with directionally consistent findings across multivariable adjustment, propensity-score methods, and the mTICI grade 2b–3 restricted analysis. The index-to-endpoint-scan interval, endpoint imaging modality, and number of evaluable post-index scans were similar between groups, reducing the plausibility that markedly differential imaging surveillance explained the finding; nevertheless, intermittent imaging could identify only the interval during which an abnormality first met the outcome criteria. After FDR correction across 17 exploratory outcomes, only new or worsening cerebral edema retained a statistical signal. Neither the 90-day 3-category mRS distribution nor mRS 0–2 showed a statistically significant between-group difference after correction, and the 95% CIs for safety risk estimates were wide.

The MARVEL trial used a fixed dose and treatment duration and evaluated the full 7-level 90-day mRS distribution as its primary clinical outcome; it found no improvement in the overall functional distribution [19]. Likewise, the 3-category 90-day mRS distribution in our study did not differ significantly between groups, and mRS 0–2 was not statistically significant after FDR correction. Thus, the 2 studies were concordant in showing no evidence of an overall long-term functional benefit, although our 3-category description was not equivalent to analysis of the full 7-level mRS. Occlusion-site findings from MARVEL suggest that the methylprednisolone signal may vary according to vascular territory, ischemic burden, and reperfusion conditions [20]. Broader evidence on glucocorticoids indicates that functional and safety effects depend on the underlying mechanism, treatment timing, and dose [21]. Our cohort was restricted to patients who had already developed END after EVT; treatment was initiated selectively after END, and the dose, initiation time, and cumulative exposure before endpoint imaging were heterogeneous. The primary outcome was a composite imaging phenotype assessed within 72 hours. These differences, together with residual confounding, may have contributed to the observed imaging association.

Adjunctive interventions targeting inflammatory signaling in the reperfusion setting can generate early biological and tissue-level signals, supporting the rationale for protection of the neurovascular unit [22]. Prethrombectomy cerebral edema is associated with greater ischemic burden and poorer outcomes [23]. Tissue recovery also depends on the speed and quality of reperfusion, and early effective perfusion restoration can complement the information provided by the final mTICI grade [24]. Imaging markers of cerebral edema capture distinct dimensions, including tissue water content, sulcal and ventricular compression, and midline shift [25]. In our study, cerebral edema was the only imaging component to retain a statistical signal after FDR correction. Hemorrhagic transformation, parenchymal hematoma, CT hyperdensity or a corresponding MRI signal abnormality, abnormal enhancement or exudative change, and midline shift did not differ significantly after multiplicity correction, suggesting that the composite association was driven primarily by edema-related phenotypes. The imaging-related management outcome of escalation of intracranial pressure therapy or decompressive craniectomy also did not remain statistically significant after correction.

Early post-EVT hemodynamic changes are associated with END and subsequent outcomes [26]. END risk assessment commonly integrates baseline neurological status, imaging extent, reperfusion status, blood pressure, and metabolic indicators [27]. Prediction tools for cerebral edema repeatedly incorporate NIHSS, ASPECTS, collateral status, and treatment time, although control of bias and external validation remain major limitations [28]. Ischemic stroke can disrupt the blood-brain barrier through tight-junction injury, altered endothelial transport, and neurovascular inflammation [29]. In our adjusted model, higher NIHSS, lower ASPECTS, poor collateral status, longer onset-to-final-angiography time, higher peak systolic blood pressure, and higher peak glucose were associated with the primary outcome. Discrimination decreased slightly after bootstrap correction and repeated cross-validation, whereas calibration metrics were close to ideal. These findings characterize internal stability; the model’s clinical predictive utility requires independent external validation.

Stress hyperglycemia is associated with tissue injury and adverse outcomes after mechanical thrombectomy [30]. Distinct post-EVT systolic blood pressure trajectories are associated with tissue and clinical outcomes [31]. Lower ASPECTS reflects a greater early ischemic burden and is associated with END and poor functional outcomes [32]. In the methylprednisolone group, baseline NIHSS was numerically higher, ASPECTS was numerically lower, poor collateral status was more common, and peak glucose was numerically higher, suggesting that treatment allocation may have been influenced by disease severity.

Propensity-score methods can improve balance in measured pre-index covariates but cannot control for undocumented or unstructured treatment-decision factors [33]. The effects of glucocorticoids on endothelial barrier function and inflammation-related gene expression depend on tissue context, dose, and duration of exposure [34]. Patients in this study generally received only a few doses before endpoint imaging, and clinical dosing regimens were heterogeneous; the observed association may therefore reflect a combination of drug effects, the natural disease course, and treatment selection.

Blood-brain barrier disruption may coexist with stroke-associated pneumonia and systemic inflammation [35]. The 95% CIs for the absolute risk differences in pneumonia, gastrointestinal bleeding, hyperglycemia, electrolyte disturbances, and other low-frequency events were wide and all crossed 0. The risk-difference estimate for hyperglycemia was positive, supporting close monitoring for hyperglycemia and infection during treatment. Dual-energy CT can improve differentiation between contrast and hemorrhagic components and offers a technical pathway for standardizing imaging outcomes in future studies [36].

### Limitations

This study has several limitations. First, the single-center retrospective design and nonprotocolized treatment assignment leave the possibility of confounding by indication, unmeasured confounding, and physician decision bias. Second, the primary outcome was a study-defined composite imaging phenotype whose components could overlap and had different clinical implications; its measurement performance requires independent external validation. Escalation of intracranial pressure therapy or decompressive craniectomy was a management outcome and should be interpreted separately. Third, patients with mTICI grade 0–2a did not achieve successful global reperfusion, limiting the mechanistic specificity of their imaging abnormalities. Fourth, contrast-related hyperdensity and early hemorrhage overlap intrinsically on conventional CT; high inter-reader agreement and the CT-specific 3-category analysis cannot fully exclude misclassification. Fifth, the first positive scan was used for outcome-positive patients and the last evaluable scan within 72 hours for outcome-negative patients, so abnormality onset was interval-censored. Although imaging timing, modality, and frequency were similar between groups, the nonstandardized imaging schedule may still have introduced informative observation bias. Sixth, the multivariable model underwent internal validation only, subgroup sample sizes were limited, and external generalizability remains uncertain. Seventh, safety event counts were small and the 95% CIs for absolute risk differences were wide.

## 5. Conclusions

In this single-center retrospective analysis, short-course methylprednisolone was associated with lower odds of the study-defined early post-EVT composite imaging abnormality, with directionally consistent findings in the mTICI grade 2b–3 restricted analysis. After FDR correction across 17 exploratory outcomes, only new or worsening cerebral edema retained a statistical signal. Neither the 90-day 3-category mRS distribution nor mRS 0–2 showed a statistically significant between-group difference after correction, and safety risk estimates were imprecise. This observational association requires further evaluation using standardized dosing, uniform imaging follow-up, and independent external validation.

## Data Availability

The datasets generated and/or analyzed during the current study are available from the corresponding author on reasonable request.
