# Supplementary material for: Association of Short-Course Methylprednisolone Therapy With Early Composite Imaging Abnormalities in Patients With Early Neurological Deterioration After Endovascular Thrombectomy for Acute Ischemic Stroke
Source: Rev Neurol. 2026 Sep 22;81(9):54438. doi: 10.31083/RN54438 (PMC13653165; doi:10.31083/RN54438)
Supplement: Supplementary file 1 [file 1576-6578-81-9-54438-s1.zip › Supplementary Material.docx]

**Supplementary Material**

**Supplementary Table 1. Admission Laboratory Variables by Treatment Group**

| Variable | Methylprednisolone group (*n* = 96) | No-methylprednisolone group (*n* = 201) | Statistic | *P* value |
| --- | --- | --- | --- | --- |
| White blood cell count, ×10⁹/L | 8.9 [7.2–11.3]; *n* = 96 | 8.5 [6.9–10.8]; *n* = 201 | *Z* = −1.52 | 0.129 |
| Neutrophils, % | 76 [68–84]; *n* = 96 | 74 [67–82]; *n* = 201 | *Z* = −1.48 | 0.139 |
| Hemoglobin, g/L | 134 ± 17; *n* = 96 | 136 ± 16; *n* = 201 | *t* = −0.97 | 0.335 |
| Platelet count, ×10⁹/L | 212 ± 66; *n* = 96 | 218 ± 70; *n* = 201 | *t* = −0.72 | 0.473 |
| Fasting glucose, mmol/L | 6.9 [5.9–8.6]; *n* = 96 | 6.7 [5.8–8.2]; *n* = 201 | *Z* = −1.14 | 0.254 |
| Glycated hemoglobin, % | 6.2 [5.7–7.0]; *n* = 93 | 6.1 [5.6–6.9]; *n* = 195 | *Z* = −0.84 | 0.401 |
| Total cholesterol, mmol/L | 4.48 ± 1.02; *n* = 96 | 4.42 ± 1.08; *n* = 201 | *t* = 0.47 | 0.642 |
| Triglycerides, mmol/L | 1.47 [1.05–2.02]; *n* = 96 | 1.42 [1.01–1.96]; *n* = 201 | *Z* = −0.61 | 0.542 |
| Low-density lipoprotein cholesterol, mmol/L | 2.73 ± 0.81; *n* = 96 | 2.67 ± 0.85; *n* = 201 | *t* = 0.59 | 0.558 |
| High-density lipoprotein cholesterol, mmol/L | 1.08 ± 0.28; *n* = 96 | 1.10 ± 0.30; *n* = 201 | *t* = −0.56 | 0.574 |
| International normalized ratio | 1.05 ± 0.14; *n* = 96 | 1.04 ± 0.13; *n* = 201 | *t* = 0.59 | 0.557 |
| Activated partial thromboplastin time, s | 31.8 ± 5.9; *n* = 96 | 31.4 ± 5.6; *n* = 201 | *t* = 0.56 | 0.579 |
| Fibrinogen, g/L | 3.4 [2.9–4.1]; *n* = 96 | 3.3 [2.8–4.0]; *n* = 201 | *Z* = −0.95 | 0.342 |
| D-dimer, mg/L fibrinogen-equivalent units (FEU) | 0.92 [0.46–1.85]; *n* = 94 | 0.86 [0.42–1.72]; *n* = 195 | *Z* = −0.74 | 0.459 |
| C-reactive protein, mg/L | 9.8 [4.2–21.7]; *n* = 93 | 8.6 [3.9–19.4]; *n* = 194 | *Z* = −1.29 | 0.197 |
| Albumin, g/L | 38.6 ± 4.2; *n* = 96 | 39.1 ± 4.5; *n* = 201 | *t* = −0.94 | 0.350 |
| Alanine aminotransferase, *U*/L | 25 ± 18; *n* = 96 | 23 ± 16; *n* = 201 | *t* = 0.93 | 0.355 |
| Aspartate aminotransferase, *U*/L | 28 ± 19; *n* = 96 | 26 ± 17; *n* = 201 | *t* = 0.88 | 0.382 |
| Creatinine, μmol/L | 76 ± 25; *n* = 96 | 74 ± 23; *n* = 201 | *t* = 0.66 | 0.509 |
| Estimated glomerular filtration rate, mL/(min·1.73 m²) | 84 ± 21; *n* = 96 | 86 ± 22; *n* = 201 | *t* = −0.76 | 0.451 |

**Note:** Data are presented as mean ± standard deviation or median [interquartile range], together with the actual nonmissing sample size (*n*). *t* indicates the independent-samples *t* test statistic; *Z*, the standardized Mann–Whitney *U* test statistic; *P*, the 2-sided *P* value; and FEU, fibrinogen-equivalent units. Each variable was compared using the available nonmissing observations.

**Supplementary Table 2. Inter-Reader Agreement for Imaging Outcomes**

| Imaging outcome | Simple agreement | *κ* (95% confidence interval [CI]) | Statistical method |
| --- | --- | --- | --- |
| Primary composite imaging outcome | 93.3% | 0.86 (0.80–0.92) | Cohen *κ* |
| Hemorrhagic transformation | 96.3% | 0.92 (0.88–0.97) | Cohen *κ* |
| European Cooperative Acute Stroke Study (ECASS) subtype | 79.1% | 0.90 (0.86–0.92) | Quadratically weighted *κ* |
| Computed tomography (CT) hyperdensity or corresponding magnetic resonance imaging (MRI) signal abnormality | 92.9% | 0.83 (0.75–0.90) | Cohen *κ* |
| New or worsening cerebral edema | 94.6% | 0.89 (0.83–0.94) | Cohen *κ* |
| Abnormal enhancement or exudative change | 93.6% | 0.82 (0.74–0.90) | Cohen *κ* |
| Midline shift | 98.3% | 0.93 (0.86–0.99) | Cohen *κ* |
| CT-specific 3-category classification (*n* = 251) | 92.8% | 0.84 (0.76–0.91) | Unweighted Cohen *κ* |

**Note:** Simple agreement is the proportion of cases for which the 2 readers assigned exactly the same category. *κ* indicates the Cohen *κ* agreement coefficient; CI, confidence interval; ECASS, European Cooperative Acute Stroke Study; CT, computed tomography; MRI, magnetic resonance imaging; and *n*, sample size. Unweighted Cohen *κ* was used for binary outcomes, quadratically weighted *κ* for ordered ECASS subtypes, and unweighted Cohen *κ* for the CT-specific nominal 3-category classification. The 95% CIs were estimated using 1,000 bootstrap resamples.

**Supplementary Table 2 (Continued). Original Pre-Adjudication Matrix for the Computed Tomography-Specific 3-Category Classification**

| Reader 1 / Reader 2 | No relevant hyperdensity | Contrast-related hyperdensity | Hemorrhagic change | Total |
| --- | --- | --- | --- | --- |
| No relevant hyperdensity | 173 | 3 | 2 | 178 |
| Contrast-related hyperdensity | 4 | 31 | 3 | 38 |
| Hemorrhagic change | 4 | 2 | 29 | 35 |
| Total | 181 | 36 | 34 | 251 |

**Note:** CT indicates computed tomography. This 3×3 matrix is based on independent readings before third-reader adjudication. The 233 observations on the main diagonal represent concordant classifications by the 2 readers; there were 5 direct disagreements between contrast-related hyperdensity and hemorrhagic change.

**Supplementary Table 3. Complete Logistic Regression Model for the Primary Composite Imaging Outcome**

| Variable | Univariable odds ratio (OR) (95% confidence interval [CI]) | *P* value | Adjusted OR (95% CI) | *P* value |
| --- | --- | --- | --- | --- |
| Short-course methylprednisolone | 0.50 (0.31–0.82) | 0.006 | 0.46 (0.27–0.80) | 0.005 |
| Age, per 10-year increase | 1.12 (0.89–1.40) | 0.327 | 1.07 (0.83–1.38) | 0.602 |
| Male sex | 0.96 (0.60–1.52) | 0.863 | 0.91 (0.55–1.52) | 0.716 |
| Baseline National Institutes of Health Stroke Scale (NIHSS), per 1-point increase | 1.08 (1.03–1.13) | 0.001 | 1.06 (1.01–1.12) | 0.027 |
| Baseline Alberta Stroke Program Early Computed Tomography Score (ASPECTS), per 1-point increase | 0.74 (0.62–0.88) | <0.001 | 0.79 (0.66–0.94) | 0.009 |
| Terminal internal carotid artery or tandem occlusion | 1.44 (0.86–2.42) | 0.167 | 1.18 (0.66–2.11) | 0.577 |
| Poor collateral status | 2.34 (1.42–3.87) | <0.001 | 1.96 (1.11–3.46) | 0.020 |
| Bridging intravenous thrombolysis | 1.23 (0.76–2.00) | 0.402 | 1.19 (0.70–2.03) | 0.522 |
| Onset-to-final-angiography time, per 60-min increase | 1.20 (1.06–1.36) | 0.004 | 1.16 (1.01–1.33) | 0.035 |
| Modified Thrombolysis in Cerebral Infarction (mTICI) grade 2b–3 | 0.49 (0.27–0.88) | 0.018 | 0.58 (0.29–1.15) | 0.121 |
| Number of thrombectomy passes, per 1-pass increase | 1.20 (1.03–1.40) | 0.020 | 1.10 (0.94–1.30) | 0.249 |
| Peak systolic blood pressure, per 10 mm Hg | 1.15 (1.04–1.27) | 0.006 | 1.12 (1.01–1.25) | 0.037 |
| Peak glucose, per 1 mmol/L | 1.13 (1.03–1.24) | 0.010 | 1.11 (1.01–1.23) | 0.038 |
| Mild related abnormality on index imaging | 1.69 (1.02–2.80) | 0.042 | 1.45 (0.84–2.51) | 0.183 |
| Any pre-index antithrombotic therapy | 1.08 (0.64–1.83) | 0.774 | 1.03 (0.59–1.80) | 0.917 |

**Note:** OR indicates odds ratio; CI, confidence interval; *P*, the 2-sided *P* value; NIHSS, National Institutes of Health Stroke Scale; ASPECTS, Alberta Stroke Program Early Computed Tomography Score; and mTICI, modified Thrombolysis in Cerebral Infarction. The multivariable logistic regression model included all variables listed in the table.

**Supplementary Table 4. Internal Validation and Exploratory Subgroup Discrimination of the Multivariable Model**

| Metric | Result (95% confidence interval [CI]) | Description |
| --- | --- | --- |
| Apparent area under the receiver operating characteristic curve (AUC) | 0.782 (0.726–0.839) | Full cohort |
| Bootstrap optimism-corrected AUC | 0.768 | 1,000 resamples |
| AUC from 10 repeats of 10-fold cross-validation | 0.754 (0.716–0.792) | Interval is the percentile interval from repeated cross-validation |
| Optimism-corrected calibration intercept | −0.03 (−0.22 to 0.16) | Ideal value, 0 |
| Optimism-corrected calibration slope | 0.94 (0.77–1.11) | Ideal value, 1 |
| Brier score | Apparent, 0.190; optimism-corrected, 0.198 | Lower values indicate less overall probability error |
| AUC for modified Thrombolysis in Cerebral Infarction (mTICI) grade 2b–3 | 0.776 (0.715–0.837) | *n* = 255; exploratory |
| AUC for mTICI grade 0–2a | 0.731 (0.568–0.894) | *n* = 42; exploratory |
| AUC for anterior-circulation occlusion | 0.785 (0.728–0.842) | *n* = 261; exploratory |
| AUC for basilar artery occlusion | 0.742 (0.582–0.902) | *n* = 36; exploratory |
| Events/model parameters | 164/15 (10.9) | Describes model complexity; not evidence of external generalizability |

**Note:** AUC indicates area under the receiver operating characteristic curve; CI, confidence interval; mTICI, modified Thrombolysis in Cerebral Infarction; *n*, sample size; and bootstrap, resampling with replacement. The Brier score evaluates overall error in predicted probabilities. Subgroup AUCs are presented for exploratory description only.

**Supplementary Table 5. Composite Outcome, Imaging Components, and Imaging-Related Management Outcome Stratified by Modified Thrombolysis in Cerebral Infarction (mTICI) Grade**

| Outcome | mTICI grade 2b–3 Methylprednisolone (*n* = 84) | mTICI grade 2b–3 No methylprednisolone (*n* = 171) | mTICI grade 0–2a Methylprednisolone (*n* = 12) | mTICI grade 0–2a No methylprednisolone (*n* = 30) |
| --- | --- | --- | --- | --- |
| Study-defined early composite imaging abnormality after endovascular thrombectomy (EVT) | 34 (40.5) | 99 (57.9) | 8 (66.7) | 23 (76.7) |
| Hemorrhagic transformation | 32 (38.1) | 72 (42.1) | 5 (41.7) | 14 (46.7) |
| Parenchymal hematoma (PH1–PH2) | 11 (13.1) | 28 (16.4) | 2 (16.7) | 6 (20.0) |
| Computed tomography (CT) hyperdensity or corresponding magnetic resonance imaging (MRI) signal abnormality | 17 (20.2) | 48 (28.1) | 4 (33.3) | 12 (40.0) |
| New or worsening cerebral edema | 21 (25.0) | 76 (44.4) | 6 (50.0) | 19 (63.3) |
| Abnormal enhancement or exudative change | 13 (15.5) | 43 (25.1) | 3 (25.0) | 9 (30.0) |
| Midline shift | 5 (6.0) | 26 (15.2) | 2 (16.7) | 8 (26.7) |
| Imaging-related management outcome: escalation of intracranial pressure therapy or decompressive craniectomy | 5 (6.0) | 24 (14.0) | 1 (8.3) | 7 (23.3) |

**Note:** Data are presented as number (%). *n* indicates sample size; EVT, endovascular thrombectomy; mTICI, modified Thrombolysis in Cerebral Infarction; PH1 and PH2, parenchymal hematoma types 1 and 2; CT, computed tomography; and MRI, magnetic resonance imaging. The mTICI grade 2b–3 analysis has greater clinical specificity, whereas the mTICI grade 0–2a analysis is exploratory and descriptive.
